# Myositis, Osteomyelitis and a Parasymphyseal Stress Fracture in a Paediatric Patient

**DOI:** 10.7759/cureus.19593

**Published:** 2021-11-15

**Authors:** Jamie Hind, Aimen Gmati, Neil Ashwood

**Affiliations:** 1 Trauma and Orthopaedics, Oxford University Hospitals National Health Service Foundation Trust, Oxford, GBR; 2 Trauma and Orthopaedics, University Hospitals of Derby and Burton National Health Service Foundation Trust, Burton, GBR

**Keywords:** dermatomyositis, anti-jo-1 antibodies, immune myositis, parasymphysis fractures, osteomyelitis diagnosis, inflammatory myositis

## Abstract

The limping child and painful hip are common presentations in many paediatric emergency units. Typically caused by mild self-limiting events, less commonly, they may be implicated in one of a group of inflammatory myopathies, or myositis. Diagnosis of this condition can be extremely difficult, and is aided by thorough clinical assessment, radiological imaging, and extensive blood serum testing. Myositis with associated osteomyelitis and a pathological fracture is an incredibly rare finding, described in this case report in a seven-year-old child.

## Introduction

Inflammatory disorders of the skeletal system are a group of rare diseases that show chronic muscular inflammation, and less often, extra-skeletal manifestations. Numerous classifications of myositis exist, determined by a careful assessment of clinical symptoms, radiological imaging and a close analysis of associated auto-antibodies [1]. Osteomyelitis is an inflammatory reaction in skeletal muscle, usually associated with bacteria, namely *Staphylococcus* as the main causative agent [2]. The combination of myositis, osteomyelitis and a stress fracture has not been documented in previous literature.

## Case presentation

**Figure 1:**
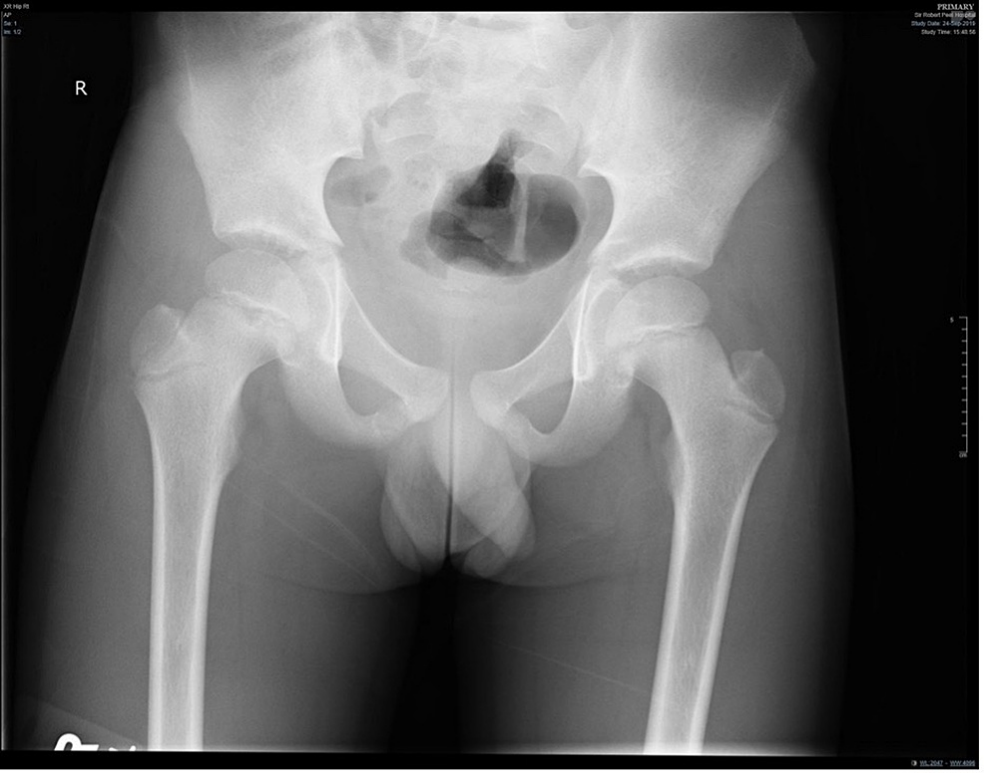
Anteroposterior pelvic radiograph showing normal pelvic anatomy, normal growth plates, and no evidence of bony injury or Perthes disease.

A seven-year old boy was seen in the emergency department with a temperature and a painful right hip. This was thought to be a result of viral arthritis or an irritable hip. The patient was discharged home and his symptoms resolved. The patient was referred to the orthopaedic team after his symptoms returned, causing him significant pain to his right hip and preventing him from attending school. Apart from eczema, he had no other medical issues. On examination he had a very slight restriction of internal rotation in the right hip when compared to the left. However, this was completely pain free. A plain radiograph of the pelvis was performed, which was normal with no evidence of bony injury or Perthes disease (Figure 1).

**Figure 2:**
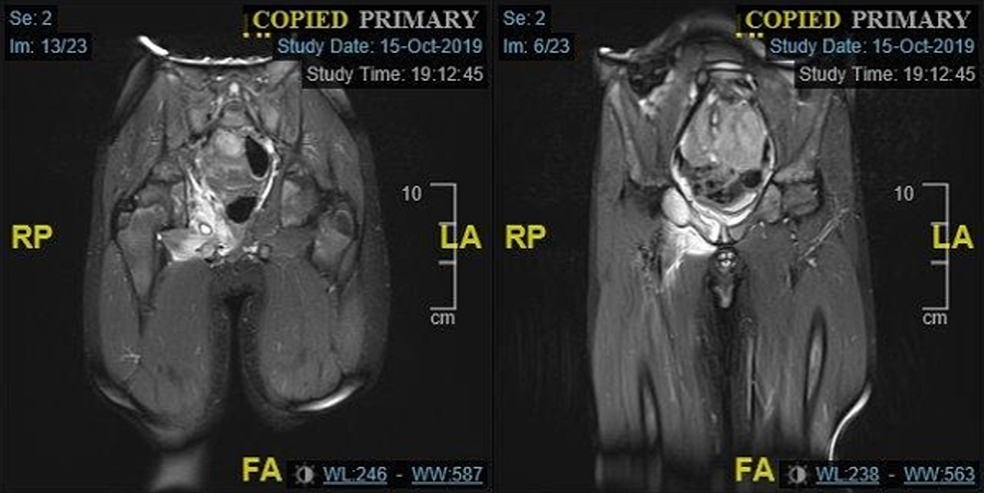
Coronal sections from T2-weighted MRI showing extensive myositis in the right obturator internus containing a collection, less severe myositis in the obturator externus and pectineus muscle (left) and suspicion of osteomyelitis of right pubic bone (right).

An MRI was requested but before this was performed the patient had another episode of right hip pain and a temperature of 38.2. He was, therefore, referred again to the orthopaedic team. On this occasion he walked into clinic with an antalgic gait. On examination he had some limitation of internal rotation compared to the left side. He had pain and tenderness around the right hip joint. He had normal neurovascular status distally. He had his MRI scan, which showed extensive myositis of the right obturator externus muscle and pectineus muscle with a small pocket of high signal collection (Figure 2). It also demonstrated osteomyelitis in the right pubic bone. He was seen in clinic and scan results explained to the patient and his family. At this time he was relatively comfortable. He had a full range of motion to the hip and there was no tenderness around the hip joint. He could straight leg raise without any problems. There were no reported spikes in temperature. He was eating, drinking and playing as normal. His white cell count and C-reactive protein were normal but erythrocyte sedimentation rate was raised slightly at 16.

**Figure 3:**
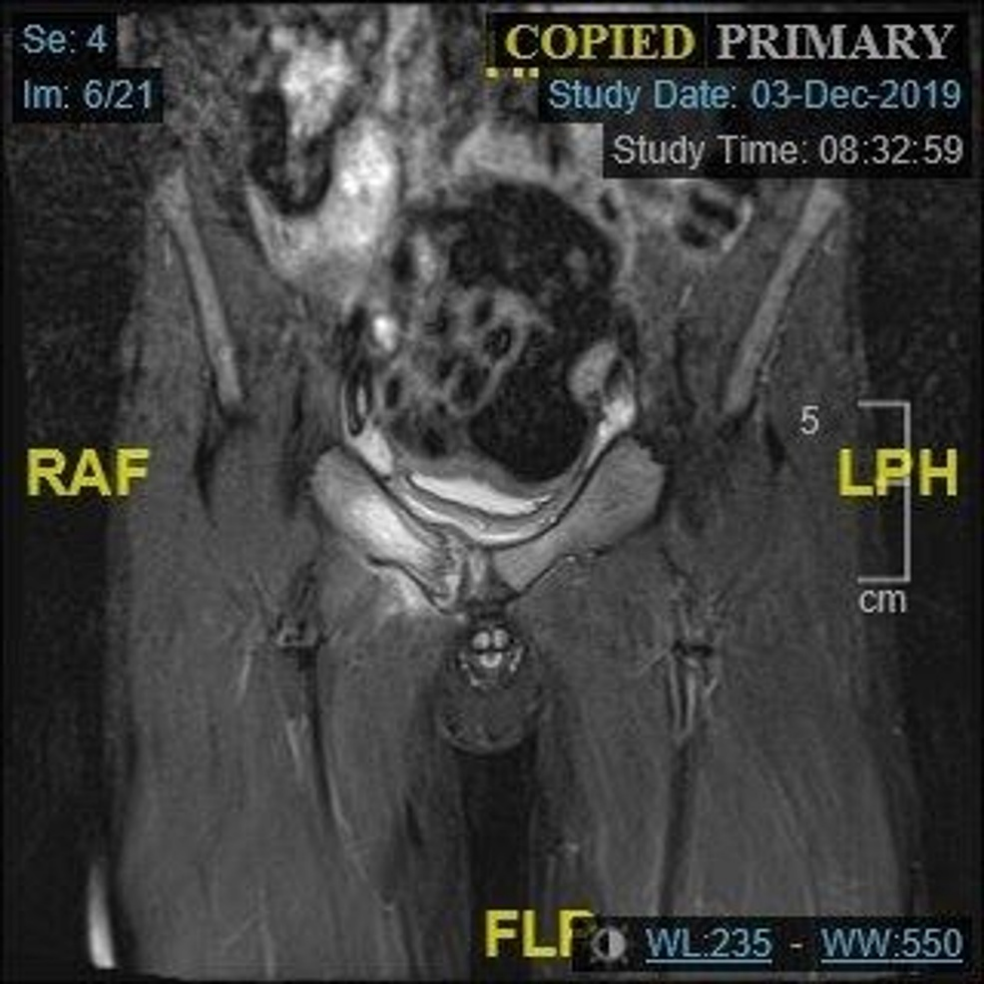
Coronal sections of T2-weighted MRI demonstrating marked oedema in the right parasymphyseal region, likely in keeping with a further area of stress reaction or stress fracture.

**Figure 4:**
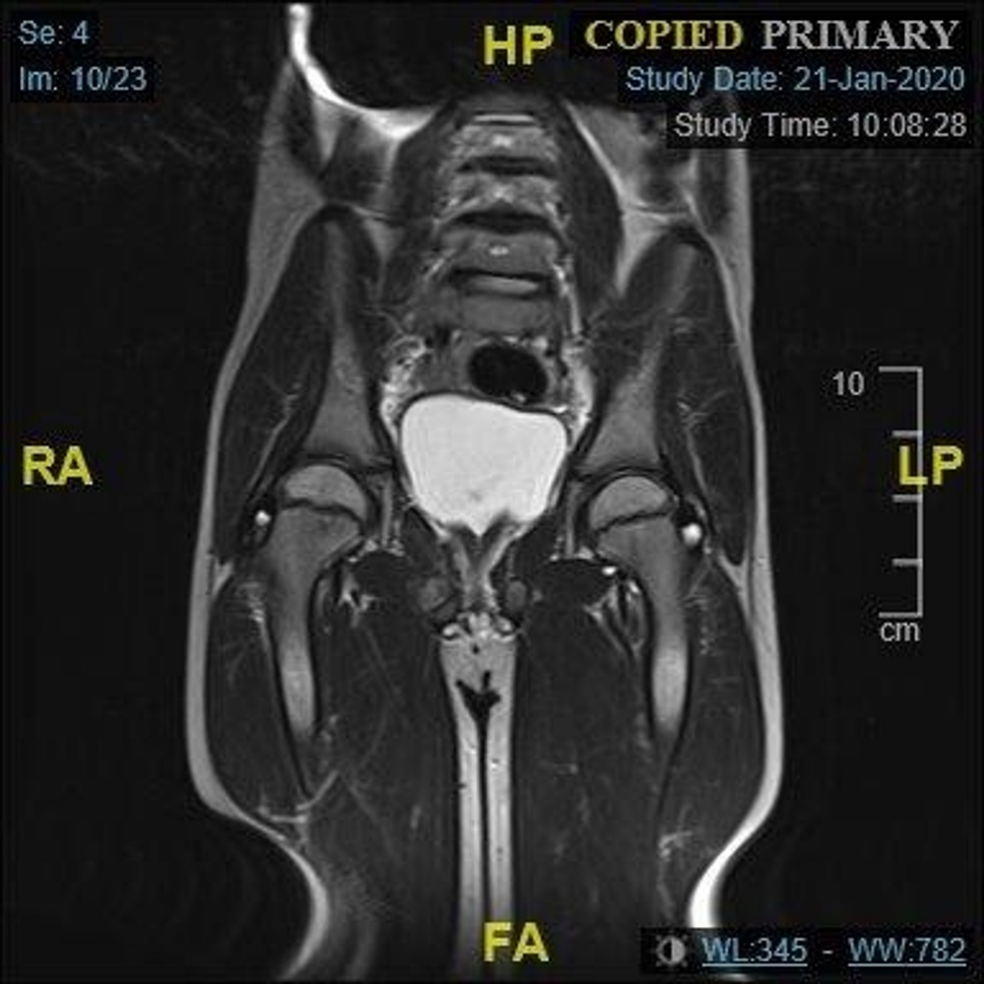
Coronal sections of T2-weighted MRI demonstrating almost complete resolution of the abnormal high signal in the right obturator internus and pectineus, and interval improvements in the inferior aspect of the left sacral ala.

A second MRI scan was performed in December, which showed significant reduction in the degree of oedema at the right ischiopubic synchondrosis and in the surrounding soft tissue, particularly abductor and lateral rotators (Figure 3). There was still marked oedema in the right parasymphyseal region likely in keeping with a further area of stress reaction or stress fracture. A further MRI was performed six weeks later (Figure 4), which showed almost complete resolution of the abnormal high signal in the right obturator internus and pectineus and interval improvements in the inferior aspect of left sacral ala. There was, however, little interval change in the oedema/inflammation in the right superior and inferior pubis rami including the synchondrosis. The patient was referred to the paediatric orthopaedic consultant. At this point, clinically he was apyrexial and clinically well. He had a normal stance and a normal gait. He had normal power in both lower limbs and a full range of movement. He had no back or hip pain. Whilst it appears that this condition has improved and may resolve, we are unable to confirm the exact aetiology and the pathophysiology.

## Discussion

Autoimmune myositis, previously known as idiopathic inflammatory myopathies, is a group of rare heterogeneous disorders characterized by chronic inflammation of skeletal muscle leading to weakness of the affected muscle(s). It can also affect other organs including skin, lungs, heart and gastrointestinal tract; however, this is less common [3]. Originally, myositis was sub-classified into polymyositis, dermatomyositis (DM) and inclusion body myositis [3]. Now it also encompasses overlap myositis [4] and necrotizing autoimmune myositis [5]. DM is an autoimmune disease that primarily involves both skin and skeletal muscles and the cutaneous manifestation in these patients may be present up to two years before any evidence of myositis occurs [6]. This is the most common sub-type of myositis [7], and unlike the other types of myositis that affect middle-aged individuals, DM predominantly affects children and adults [8]. The cutaneous manifestations of DM are well documented and some of the more specific skin lesions include Gottrons papules, violaceous scaling papules on the skin overlying the joints and proximal nail folds; Gottrons sign, violaceous patches overlying the knees; ‘V’ sign, confluent macular erythema over the lower anterior neck and upper anterior chest; and the Shawl sign, erythema over the upper back, posterior neck, and shoulders and lateral arms [9]. Other skin manifestations include telangiectasia, erythema over the elbows and knees, dystrophic cuticles and calcinosis [10]. The above skin lesions may be worsened by exposure to ultraviolet (UV) A and UVB light [6]. Interstitial lung disease is an important associated condition in up to 40% patients with DM causing significant morbidity and mortality to these patients [11]. There is no set diagnostic criteria for myositis, so it is often a clinical diagnosis based on history, examination and investigation findings. 

The history of DM is often of proximal muscle weakness initially, with or without myalgia or tenderness and the skin lesions as mentioned previously [12]. On examination the patient experiences pain when the affected muscles are stretched or contracted or the affected muscle is palpated. Blood test reveals elevated enzyme levels, most notably creatine kinase. MRI of the muscle and electromyography are also used in the work-up for patients with suspected myositis as well as detailed histopathological examination of a biopsy of the skeletal muscle [5]. Myositis-specific autoantibodies are detected to help diagnose myositis and also to help identify the clinical sub-group [13]. The Jo-1 autoantibody is the most common [14]. Treatment is usually initiated with intravenous glucocorticosteroids, followed by oral corticosteroid with a tapering regime [15]. Our patient, however, did not receive any treatment. Myositis in combination with osteomyelitis is a rare phenomenon and has only been reported in a few case reports: One in a patient with a *Pasteurella multocida* infection [16], one in a patient following a course of radiotherapy [17], and one in a patient with chronic recurrent multifocal osteomyelitis [18]. This case is unlike the aforementioned, as no cause could be identified. Whilst it may be possible that his eczema predisposed him to myositis and osteomyelitis, equally, this may have been a cutaneous manifestation of myositis not previously documented. In addition to this, this case report is also unique in that the patient had an associated pathological stress fracture. The combination of myositis, osteomyelitis and an associated stress fracture has not been documented previously [19]. Whilst it has been noted that pathological stress fractures are a recognized but rare complication of osteomyelitis [20], its association with myositis is less well documented. 

## Conclusions

Autoimmune myositis is a rare condition associated with significant morbidity. DM is the most common subtype of this condition, especially in children. Myositis with associated osteomyelitis is a particularly rare finding. This case report is of a patient with an atypical presentation of myositis, with associated osteomyelitis and a pathological stress fracture. 
